# Bifunctional Carbon Dots—Magnetic and Fluorescent Hybrid Nanoparticles for Diagnostic Applications

**DOI:** 10.3390/nano10071384

**Published:** 2020-07-16

**Authors:** Ilana Perelshtein, Nina Perkas, Shai Rahimipour, Aharon Gedanken

**Affiliations:** Department of Chemistry, Bar-Ilan Institute of Nanotechnology and Advanced Materials, Bar-Ilan University, Ramat-Gan 5290002, Israel; ilana.perelshtein@biu.ac.il (I.P.); nina.perkas@biu.ac.il (N.P.); Shai.rahimipour@biu.ac.il (S.R.)

**Keywords:** bifunctional nanoparticles, magnetic, fluorescent, C-dots, air-stable Fe, ultrasound

## Abstract

There is a huge demand for materials capable of simple detection or separation after conjugation with specific biologic substances when applied as a diagnostic tools. Taking into account the photoluminescence properties of C-dots and the highly magnetic properties of Fe(0), a new hybrid composite of these components was synthesized via ultrasound irradiation. The material was fully characterized by various physicochemical techniques. The main goal of the current study was to obtain a highly magnetic and intense fluorescent hybrid material. The goal was achieved. In addition, magnetic particles tended to agglomerate. The new hybrid can be suspended in ethanol, which is an additional feature of the current research. The dispersion of the hybrid nanoparticles in ethanol was achieved by utilizing the interaction of iron particles with C-dots which were decorated with functional groups on their surface. The newly formed hybrid material has potential applications in diagnostic by conjugating with specific antibodies or with any other biologic compounds. Such application may be useful in detection of various diseases such as: cancer, tuberculosis, etc.

## 1. Introduction

The ability of magnetic nanoparticles to be directed to a specific organ in the human body by an external field makes these particles very viable for biomedical applications [1,2,3]. Among all the magnetic nanomaterials, iron and iron oxides are the most frequently used materials due to their nontoxicity and relative biocompatibility. Moreover, Fe_2_O_3_ is the one of few inorganic compounds approved by Federal Drug Administraton (FDA) for bioimaging [4]. At the same time, the stability and biocompatibility of magnetic nanoparticles (NPs) may be significantly improved by coating with either inorganic (silica, hydroxyapatite) or organic (chitosan, collagen, polyethylene glycol) materials to modify their surface [5,6,7]. Recently, carbon dots (C-dots) have attracted considerable interest because of their wide range potential applications in different areas of research, due to the superior optical properties, excellent biocompatibility, small size and low cost of production [8,9,10].

Several studies have been report combining C-dots with magnetic nanomaterials and developing multifunctional systems for biomedical application. For example, the combination of magnetite Fe_3_O_4_ nanocrystals and fluorescent C-dots in porous carbon was synthesized by the solvothermal method [11]. The carbon/Fe_3_O_4_ hybrid C-dots with oligomers of ethylene glycol or polyethyleneimine for surface functionalization and passivation have been prepared in a thermal carbonization synthesis using microwave energy, coupled with magnetic separation [12]. The magnetic hybrid -C-dots synthesized by the microwave method revealed fluorescence over the visible range, but the fluorescence quantum yield has been found to be lower than that of the neat C-dots. The low emission intensity was a result of a possible quenching effect due to the presence of magnetite in the dot structure [13]. Such magnetite-carbon dots hybrid nanostructured demonstrated great promise for simultaneous imaging diagnostics and high efficacy therapy. In most of the studies nanostructured magnetite Fe_3_O_4_ was applied as source of magnetic properties for nanohybrid materials, despite its relatively low saturation of magnetization [11,12,13,14,15,16].

Recently, several other publications reported the facile fabrication of Fe (II) [17,18] or Fe (III) doped C-dots [19] by the hydrothermal method. The as-synthesized Fe ions doped C-dots showed bright blue fluorescence and good biocompatibility that can be successfully used for bioimaging. Xiao and coworkers reported on bifunctional material, fluorescent and magnetic N,Co–C-dots for application as a probe for cholesterol and uric acid detections in human blood serum [20]. The N,Co–C-dots exhibited good ferromagnetic and excellent optical properties even under extremely harsh environmental conditions. The fluorescent probe was successfully utilized for the determination of cholesterol and uric acid in human blood serum with satisfying results. To the best of our knowledge there is still no report in the scientific literature on the synthesis or application using metallic iron nanoparticles (Fe(0)) combined with fluorescent C-dots. Such a hybrid is expected to demonstrate better magnetic properties than the composites prepared to date.

Earlier we reported a unique method for the preparation of air stable iron (ASFe) NPs using the power ultrasound method [21]. After thermal annealing in argon at 750 °C the nanocrystalline iron of 150 nm in size demonstrated strong ferromagnetic properties. The air stability of ferromagnetic iron NPs was explained due to the formation of interfacial iron carbide layer during annealing. Recently, we performed a facile synthesis of highly fluorescent C-dots from polyethylene glycol (PEG-400) in a one-step sonochemical approach [22].

The motivation behind the current research is to develop a bioimaging material having two functions. On one hand, the material is fluorescent; on the other hand, the material may be attracted to an external magnet. Such a bifunctional material is expected to conjugate with some specific antibodies and applied as diagnostic tools for detection of specific diseases, such as lung cancer. For example, for detection of lung bacterial diseases or lung cancer, MPO, TNF-α and IL-8 are biomarkers commonly analyzed in sputum indicating the presence of a bacterial infection. Commercially available polyclonal antibodies can be labeled with colored redox active molecules for the detection of TNF-α, IL-8, MPO and exosomes. One of the advantages of capping Fe(0) with C-dots is the fact that carbon dots are surrounded by many functional groups such as hydroxyl, carboxylic groups that may be crucial for further conjugation of the magnetic particles to biologic substances. In addition, superparamagnetic iron nanoparticles are hardly dispersed in aqueous medium which may makes it difficult to apply such nanoparticles as diagnostic tools. The presence of C-dots in the hybrid material may increase the dispersity of the highly magnetic iron. The current study reports on a formation of hybrid nanomaterial based on nanocrystalline zero-valent iron and C-dots using the sonochemical method.

## 2. Experimental

### 2.1. Synthetic Procedure

Air stable iron (ASFe) was synthesized from iron pentacarbonyl [Fe(CO)_5_] of 99.5% purity purchased from STREM Co (Newburyport, MA 01950-4098 USA). Polyethylene glycol 400 (PEG-400) of analytical grade was purchased from Sigma–Aldrich (Antwerp, Belgium). All materials were used without further purification.

The synthesis of ASFe was performed according to the procedure published in [21]. Namely, a 1-M solution of Fe(CO)_5_ in diphenyl methane was sonicated for 3 h under argon with immersed Ti–horn of 750 W sonogenerator model VCX, frequency 20 kHz (Sonics and Materials Inc., Newtown, CT 06470, USA). Temperature was kept at −10–0 °C during sonication by a JULABO FT-901 cooler (JULABO LABORTECHNIK, GmbH, Seelbach, Germany). The product was separated by centrifugation, washed 3 times with pentane inside a N_2_-filled glove box and dried under vacuum at room temperature. The obtained solid was annealed at 750 °C for 3.5 h under Ar flow.

The sonochemical preparation of C-dots is described in [22]. The process was as follows: 12 mL of polyethylene glycol (PEG-400) was put into a glass tube and sonicated with immersed Ti-horn with a frequency of 20 kHz for 2.5 h. The reaction temperature was kept at 55 °C using a water bath. The obtained product was chilled to room temperature and kept in a dark place.

The Fe@C-dots were prepared by the following procedure: 3 mL of C-dots synthesized as described above were diluted with 22 mL ethanol and treated with ultrasonic bath (MRC ACP-120H) for 15 min. Then 100 mg of ASFe powder was added to the slurry (content of ASFe in the reaction slurry corresponded to 4 mg/mL) and sonicated with the immersed Ti-horn for 30 min keeping the temperature of the solution at 50 °C by placing the reaction cell into a water bath. The product was marked as Fe@C-dots and kept in a dark place. For the X-ray diffraction (XRD) and magnetic measurements the suspension of Fe@C-dots was diluted with ethanol at 1:1 *v/v* ratio, treated in the ultrasonic bath for 30 min, centrifuged, and the obtained powder was dried under vacuum overnight.

### 2.2. Characterization Technique

The structural analysis was performed by XRD measurements on a Bruker D8 diffractometer (Bruker AXS GmbH, Karlsruhe, Germany) with Cu Kα radiation. The product morphology was studied by high resolution transmission electron microscopy (HRTEM) on a JEOL 2100 microscope, operated at accelerating voltage of 200 kV. Magnetic characterization of the dry phase was performed at room temperature using a Quantum Design MPMS SQUID magnetometer (Quantum Design, San Diego, CA 92121, USA). The error margin in weighing the samples for magnetic measurements did not exceed 0.5%. The fluorescence of the C-dots was measured by a fluorescence spectrophotometer (Varian Cary Eclipse, Varian GmbH Darmstadt, Germany). The FTIR analysis was carried out on Bruker Tensor 27 (Bruker Optik GmbH, Ettlingen, Germany) device with a single reflection diamond ATR assessor. The chemical composition was analyzed by inductive coupled plasma–atomic emission spectroscopy on an ICP-spectrometer ULTIMA 2501 JOBINYVON (Horiba Reichenbach, Germany).

## 3. Results and Discussion

One of the aims of the current research is to optimize conditions for creating the bifunctional material and maximize the physical properties of the hybrid in terms of fluorescence and magnetization. The ratio between C-dots and iron nanoparticles was varied for obtaining a hybrid material with the desired properties. Moreover, the concentration of the reactants was also changed till the optimized value was achieved. Reaction time was modified from 15 min to 60 min. The optimal parameters for synthesizing a hybrid bifunctional nanomaterial, magnetic and fluorescent, are described in the experimental part. In addition to forming a bifunctional material, the hybrid structure was easily dispersed in ethanol. The dispersion was stable for a few days. The latest is an advantage when such magnetic compounds should be applied in a diagnostic device. Pure iron nanoparticles were barely suspended in a solvent due to the high attractive forces and agglomeration. The incorporation of C-dots allowed the material to be more dispersible.

### 3.1. Structural Characterization

#### 3.1.1. XRD

**Figure 1:**
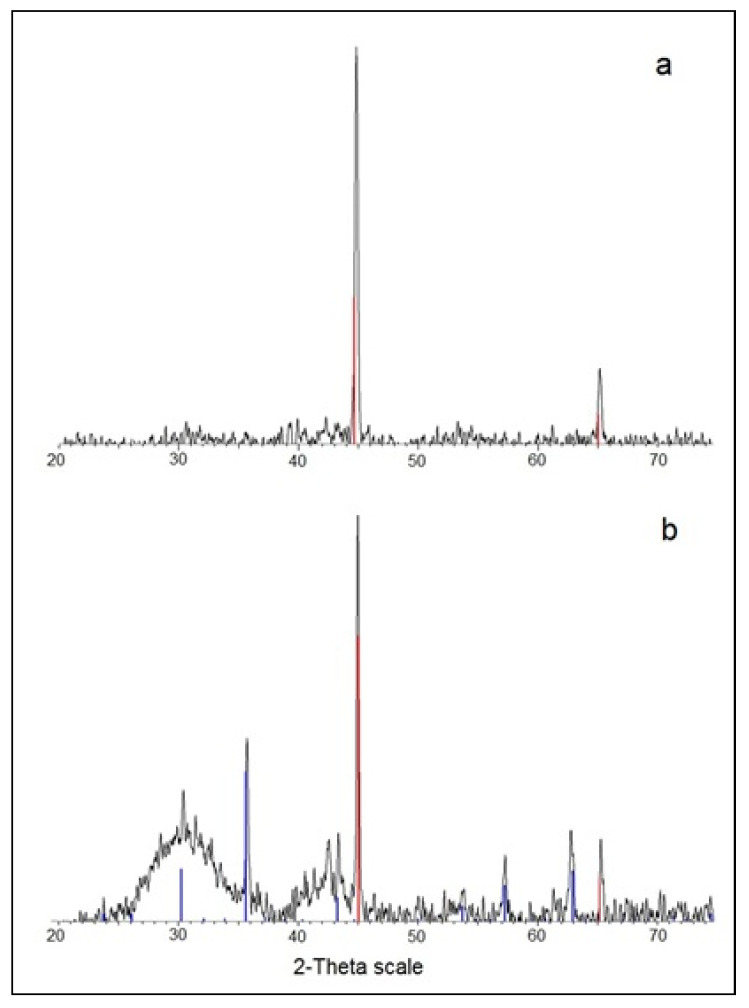
XRD patterns of ASFe. (**a**) As prepared, (**b**) after modification with C-dots.

The sonochemically synthesized ASFe NPs after annealing were examined by XRD, and the obtained pattern (Figure 1a) indicates the formation of a pure Fe(0) phase. The peaks at 44.7° and 65.0° are ascribed to the (110) and (200) planes, respectively, of the cubic Fe by comparison with the JCPDS card file no. 06-0696. There was no indication to the formation of an oxidized phase of iron. After modification with C-dots, in addition to the Fe(0), an oxide phase of maghemite Fe_2_O_3_, with diffraction peaks at 30.5°, 35.8°, 43.5°, 57.4° of 206, 313, 012, 214 planes corresponding to JCPDS 39-1346 was observed as well (Figure 1b). The metallic core of magnetic nanoparticles may be passivated by gentle oxidation upon the exposure to the solvent during the sonication with C-dots that may explain the appearance of the iron oxide phase. Still the strongest diffraction peaks were those of elemental iron.

#### 3.1.2. Morphology and Composition

**Figure 2:**
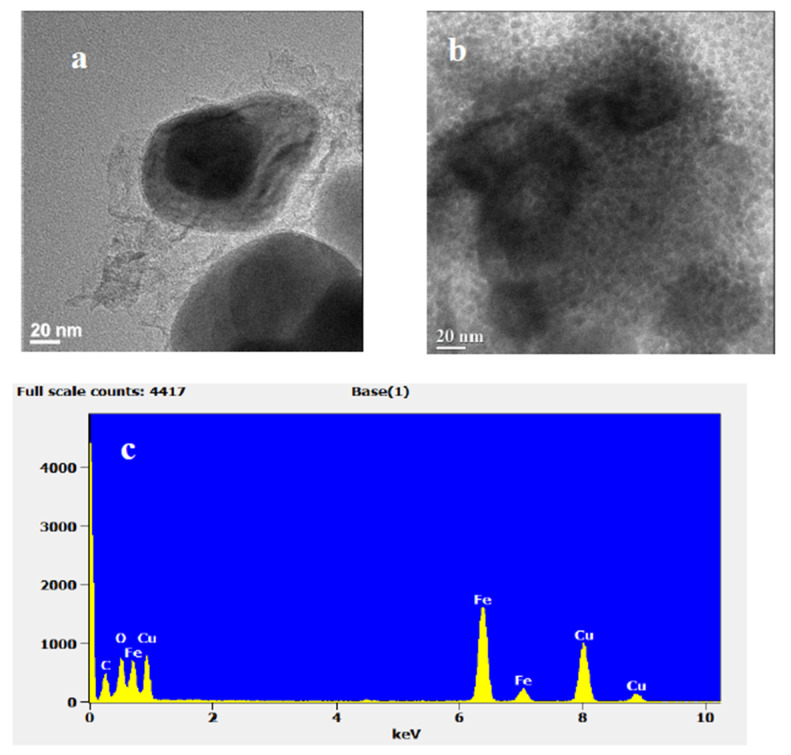
HRTEM images and EDS of ASFe. (**a**) As prepared; (**b**) after modification with C-dots; (**c**) EDS spectra of Fe@C-dots hybrid.

The morphology of Fe@C-dots, was studied by HR TEM; the images are presented in Figure 2. The particle’s size of the as prepared ASFe was estimated as 150 nm. As it is depicted on Figure 2a, the iron is surrounded with a carbon layer, which protects the particles from oxidation. After the modification of ASFe with C-dots, the metal nanoparticles are covered with very small C-dots nanoparticles of about 5 nm in size corresponding to the size of the sonochemically prepared C-dots (Figure 2b) [20]. The energy dispersive spectrum (EDS) of the local region (Figure 2c) confirms the presence of Fe in the Fe@C-dots hybrid. The total content of iron phase in the Fe@C-dots corresponds to 62.9 wt% according to the ICP analysis.

#### 3.1.3. Magnetic Properties

**Figure 3:**
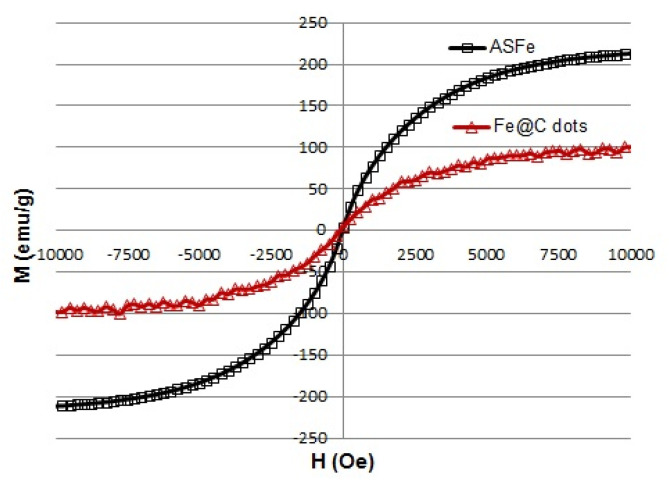
Magnetization curves of air stable iron and its hybrid with C-dots.

Magnetization curves of the products are illustrated in Figure 3. The ASFe NPs demonstrated superparamagnetic behavior with the saturation magnetization (M_s_) of ~200 emu/g. The Fe@C-dots hybrid was also superparamagnetic with a saturation magnetization of 100 emu/g that was significantly higher than that which was published for the same hybrid of magnetic particles used for the biomedical application [11,16]. Such a high value was required to ensure the effective trapping of the magnetic nanoparticles by a NdFeB magnet used in diagnostic devices.

### 3.2. Photoluminescence (PL) Properties of Fe@C-Dots

**Figure 4:**
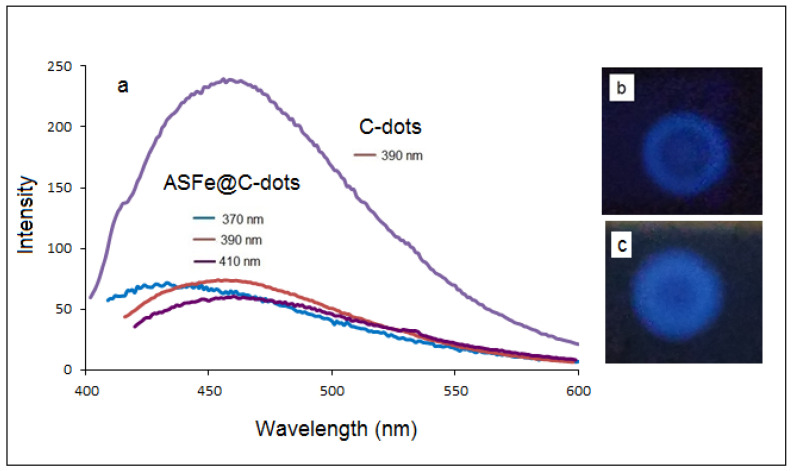
(**a**) Fluorescence of Fe@C-dots obtained with different excitation wavelengths compared with synthesized C-dots; (**b**) photograph of the Fe@C-dots drop under UV light (365 nm); (**c**) photograph of the Fe@C-dots drop under UV light (365 nm) after 3 months.

The fluorescence emitted from the Fe@C-dots dispersion was recorded at different excitation wavelengths (370, 390 and 410 nm); the spectra are presented in Figure 4a. Despite the reduced intensity, the fluorescent of the hybrid material was in a good correlation with the fluorescent properties of bare C-dots as previously published [22]. The emission spectra demonstrated broad bands centered between 440 and 480 nm. The maximum emission intensity was obtained at the excitation wavelength of 390 nm. Figure 4b demonstrated photoluminescence of a drop of Fe@C-dots dispersion on an alumina TCL plate after exposure to UV light of 365 nm.

### 3.3. FTIR Analysis

**Figure 5:**
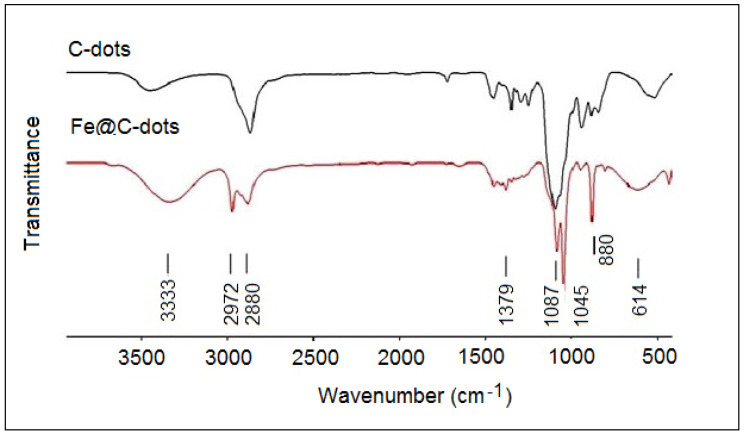
FT IR spectra of C-dots and of Fe@C-dots.

Figure 5 displays the FTIR spectrum of Fe@C-dots in comparison with the bear C-dots prepared by the sonication method. The starting materials—C-dots and ASFe—had active groups on the surface. Hydroxyl and carboxyl groups appear on the surface of carbon dots, while the air-stable iron was surrounded by a carbon layer. The IR spectrum of C-dots was very similar to that of PEG 400, which was used as raw material for the synthetic procedure [23]. The broad absorption at about 3500 cm^−1^ in the C-dots spectrum was due to the stretching vibrations of intermolecular bonded O–H groups. For the Fe@C-dots the absorption in this region was more intensive and was blue shifted to 3333 cm^−1^. This was probably because of the formation of additional hydrogen bonds with ethanol which occurred during the synthesis of Fe@C-dots. The band near 2880 cm^−1^ in both samples was due to the aliphatic C–H stretching. In the Fe@C-dots, a new band appeared at 2972 cm^−1^; it was assigned to the methoxy group [24]. The bands in the region of 1400–1200 cm^−1^ were due to C–H bending vibrations and to the combination of O–C–H and C–O–H deformation bands as it also observed for PEG [25]. The peaks at 1087 and 1046 cm^−1^ were attributed to the C–O stretch vibrations [24,25]. The sharp band at 880 cm^−1^ and broad band at 614 cm^−1^ in the hybrid spectra could be assigned to Fe–O bending and stretching vibrations, correspondingly, according to the references [26,27]. Thus, based on the FTIR analysis it was proven that the C-dots were associated with iron nanoparticles through the oxygen atoms on the surface. Obviously—despite the neutrally charged ASFe—after their interaction with the C-dots at 55 °C for 0.5 h at ambient atmosphere, the iron was partially oxidized and formation of the Fe@C-dots hybrid took place through the oxidized centers. The presence of iron oxide phase was also observed by XRD (Figure 1b).

It should be mentioned that the magnetic and fluorescent properties of the Fe@C-dots were controlled for 3 months and no changes were observed. This indicates on a good stability of the hybrid.

## 4. Conclusions

The iron–carbon dots nanohybrid—Fe@C-dots—was prepared for the first time using a facile ultrasound assisted procedure. The precursors—air stable iron nanoparticles and C-dots—were also synthesized by the sonochemical method. The structure of Fe@C-dots was found as round shape iron NPs of 150 nm, decorated with highly dispersed carbon dots. The product exhibited large enough magnetic and fluorescent properties. The formed hybrid nanomaterial was dispersed in ethanol providing a strong advantage enable applying it as a detection tools where the flow of the sensor was crucial. The fact that C-dots are decorated with functional groups allows further conjugating of Fe@C-dots with antibodies for targeting specific cells. The reported compound could serve as a potential candidate for bioimaging applications and in diagnostic devices based on magnets.
